# Characterizing the bioburden of ESBL-producing organisms in a neonatal unit using chromogenic culture media: a feasible and efficient environmental sampling method

**DOI:** 10.1186/s13756-021-01042-2

**Published:** 2022-01-24

**Authors:** Moses Vurayai, Jonathan Strysko, Kgomotso Kgomanyane, One Bayani, Margaret Mokomane, Tichaona Machiya, Tonya Arscott-Mills, David M. Goldfarb, Andrew P. Steenhoff, Carolyn McGann, Britt Nakstad, Alemayehu Gezmu, Melissa Richard-Greenblatt, Susan Coffin

**Affiliations:** 1 Department of Medical Laboratory Sciences, Faculty of Health Sciences, University of Botswana, Gaborone, Botswana; 2 Department of Paediatric & Adolescent Health, Faculty of Health Sciences, University of Botswana, Gaborone, Botswana; 3 Global Health Center, Children’s Hospital of Philadelphia, Philadelphia, USA; 4 Botswana-UPenn Partnership, Gaborone, Botswana; 5 Ministry of Health and Wellness, Gaborone, Botswana; 6 Department of Pathology and Laboratory Medicine, University of British Columbia, Vancouver, Canada; 7 Perelman School of Medicine, University of Pennsylvania, Philadelphia, USA; 8 Division of Paediatric and Adolescent Medicine, Institute of Clinical Medicine, University of Oslo, Oslo, Norway

**Keywords:** Neonatal sepsis, Extended-Spectrum-Beta-Lactamase, Enterobacterales, Pseudomonadales *Klebsiella*, *Acinetobacter*, Bioburden, Environmental sampling, Hand hygiene, Powdered Infant Formula, Infection control, Chromogenic culture media

## Abstract

**Introduction:**

Infections due to extended spectrum beta-lactamase producing organisms (ESBL) have emerged as the leading cause of sepsis among hospitalized neonates in Botswana and much of sub-Saharan Africa and south Asia. Yet, ESBL reservoirs and transmission dynamics within the neonatal intensive care unit (NICU) environment are not well-understood. This study aimed to assess the efficiency and feasibility of a chromogenic-culture-media-based environmental sampling approach to characterize the ESBL bioburden within a NICU.

**Methods:**

A series of four point-prevalence surveys were conducted at a 36-bed NICU at a public tertiary referral hospital in Botswana from January-June 2021. Samples were collected on 4 occasions under semi-sterile technique using 1) flocked swabs & templates (flat surfaces); 2) sterile syringe & tubing (water aspiration); and 3) structured swabbing techniques (hands & equipment). Swabs were transported in physiological saline-containing tubes, vortexed, and 10 µL was inoculated onto chromogenic-agar that was selective and differential for ESBL (CHROMagar™ ESBL, Paris, France), and streaking plates to isolate individual colonies. Bacterial colonies were quantified and phenotypically characterized using biochemical identification tests.

**Results:**

In total, 567 samples were collected, 248 (44%) of which grew ESBL. Dense and consistent ESBL contamination was detected in and around sinks and certain high-touch surfaces, while transient contamination was demonstrated on medical equipment, caregivers/healthcare worker hands, insects, and feeding stations (including formula powder). Results were available within 24–72 h of collection. To collect, plate, and analyse 50 samples, we estimated a total expenditure of $269.40 USD for materials and 13.5 cumulative work hours among all personnel.

**Conclusions:**

Using basic environmental sampling and laboratory techniques aided by chromogenic culture media, we identified ESBL reservoirs (sinks) and plausible transmission vehicles (medical equipment, infant formula, hands of caregivers/healthcare workers, & insects) in this NICU environment. This strategy was a simple and cost-efficient method to assess ESBL bioburden and may be feasible for use in other settings to support ongoing infection control assessments and outbreak investigations.

## Introduction

In recent decades, infections due to multidrug resistant (MDR) enteric organisms have emerged as the leading causes of neonatal sepsis in sub-Saharan Africa and south Asia [1–3]. In Botswana, the most common causes of laboratory-confirmed bloodstream infection (BSI) among hospitalized neonates are Gram-negative bacteria, most commonly *Klebsiella* species [4]. Over 80% of neonatal bloodstream *Klebsiella* isolates in this setting are reported to be extended spectrum beta-lactamase producing 1[4]. Carbapenem resistance is also emerging as a cause of neonatal sepsis in southern Africa [5, 6]. Infections due to carbapenem-resistant and extended spectrum beta-lactamase producing organisms (ESBL) are difficult to treat and confer a high mortality risk in neonates; over one in three neonates with an ESBL bloodstream infection will die [1, 7].

Hyper-endemic rates of neonatal sepsis in sub-Saharan Africa and south Asia are thought to be driven by overcrowded neonatal wards, barriers to effective hand hygiene, equipment re-use, and limited laboratory capacity to detect and respond to outbreaks. Infection prevention efforts are often thwarted because the ecology and transmission dynamics of these organisms within the neonatal intensive care unit (NICU) are not well understood. Bacteria in the Enterobacterales (e.g. *Klebsiella*, *Enterobacter* spp.) and Pseudomonadales (e.g. *Acinetobacter, Pseudomonas* spp.) orders are well suited to survive in moist and warm settings and emerging data suggest that damp reservoirs within the hospital environment, such as sink drains, washbasins, and oxygen humidifiers, could contribute to the acquisition of ESBL among patients [8–10]. However, little is known about potential ESBL reservoirs specifically within NICUs and whether they contribute to neonatal colonization and disease.

Environmental surface sampling in healthcare facilities is warranted to identify reservoirs and vehicles for clinically important pathogens [11]. However, infection control teams in resource-limited settings often lack the conventional equipment (e.g. sponge sticks, extractors, etc.) and laboratory capacity to conduct a meaningful investigation. Moreover, environmental sampling data can be difficult to interpret and are often skewed toward recovery of Gram positive organisms with unclear clinical relevance [12].

Environmental sampling techniques can be classified based on: a) sampling device (e.g. swab vs. sponge), b) method of sample preparation (e.g. dry vs. pre-moistened), c) transport and storage method, and d) sample processing (e.g. extraction, enrichment, incubation) [13]. Techniques that seek to identify specific pathogens typically focus on demonstrating qualitative presence of organisms of interest, whereas quantitative data are needed to characterize microbial bioburden to target interventions to the reservoirs with the greatest burden of potentially pathogenic organisms [13]. Environmental sampling in NICUs has historically focused on qualitative identification of pathogens as part of an outbreak investigation or as part of specific quality assurance purposes [14]. Reports of persistent reservoirs (as opposed to transient contamination) for ESBL identified within a NICU are rare [15].

In this study, we aimed to assess the efficiency and feasibility of a chromogenic-culture-media-based environmental sampling approach using basic sample collection equipment in a NICU where infections with ESBL are hyper-endemic. By deploying this technique over time on a range of samples from water, surfaces, hands and equipment in the hospital environment, we aimed to characterize the ESBL bioburden, and identify reservoirs and vehicles, which could then be targets for remediation and disinfection.

## Methods

### Study design

This study consisted of a series of point prevalence surveys, which took place on four separate occasions from January-June 2021. It underwent ethical review and approval by the Health Research and Development Committee at Botswana’s Ministry of Health and Wellness and the Institutional Review Boards at University of Botswana and the healthcare facility whether this study was carried out.

### Study setting

The study was conducted in a 36-bed NICU within a 530-bed public tertiary referral hospital in Botswana where over 8000 deliveries occur annually. The neonatal unit is a one-storey block covering a total of 315 square meters, of which 87 square meters are patient care areas (average of 2.5 m^2^ per patient-mother dyad). The most common diagnoses among patients in this NICU are prematurity-related complications, hypoxia-related injuries, and sepsis. Neonatal care is provided by approximately 16 healthcare workers per day shift, 4–6 of whom are nurses. Neonatal care includes: oxygen/ventilatory support, cardio-respiratory monitoring, enteral and parenteral nutrition, thermoregulation, transfusion, post-surgical care, phototherapy, and fluid/electrolyte management. Because of the shortage of staff, enteral feeding (both oral and gavage) is administered primarily by caregivers (mainly mothers). The unit’s doors and windows open directly outdoors, which episodically results in entrance by flies and cockroaches, and has prompted the use of Light Emitting Diode (LED) insect traps.

### Environmental sampling technique

**Fig. 1:**
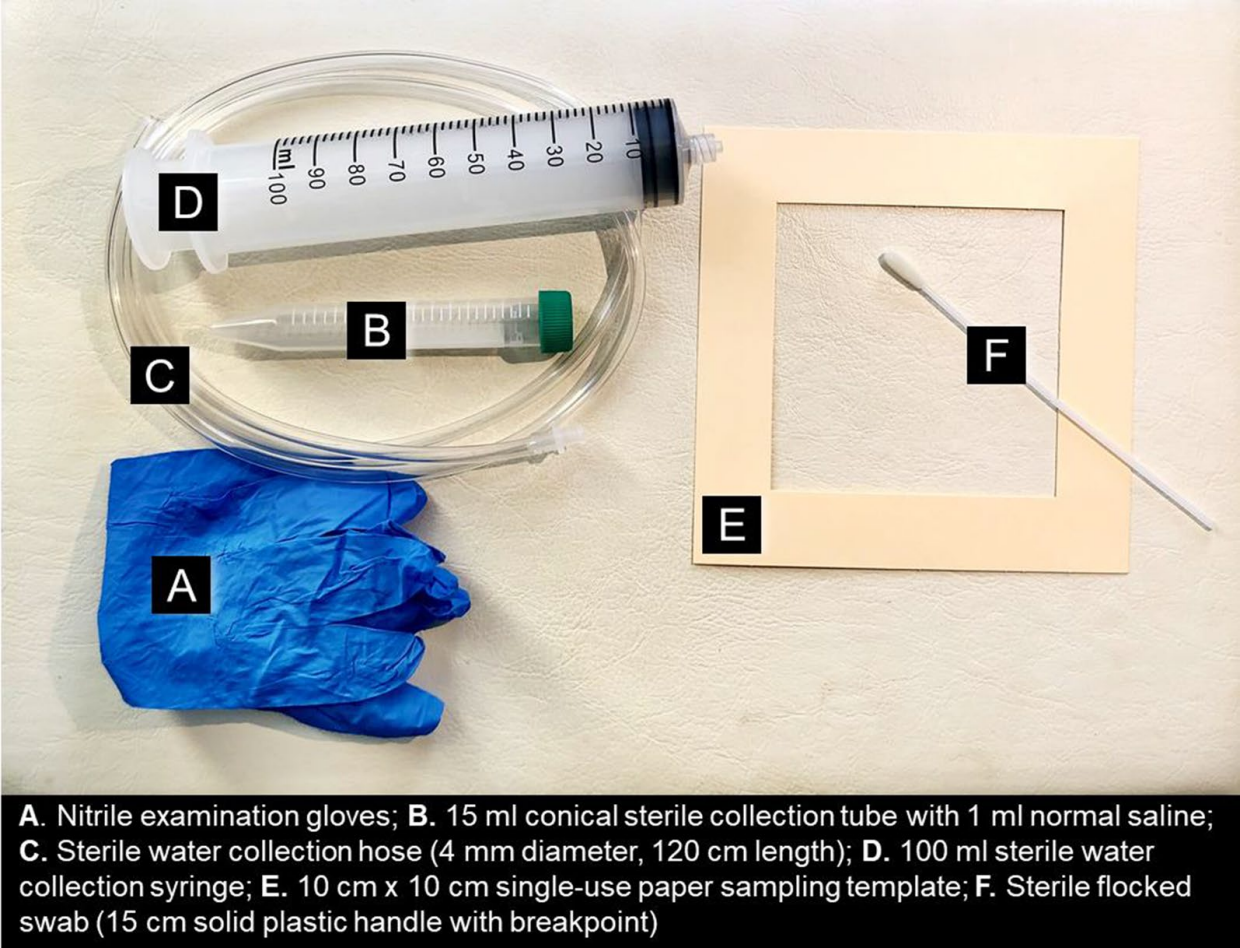
Devices used for environmental sampling in the neonatal unit

**Fig. 2:**
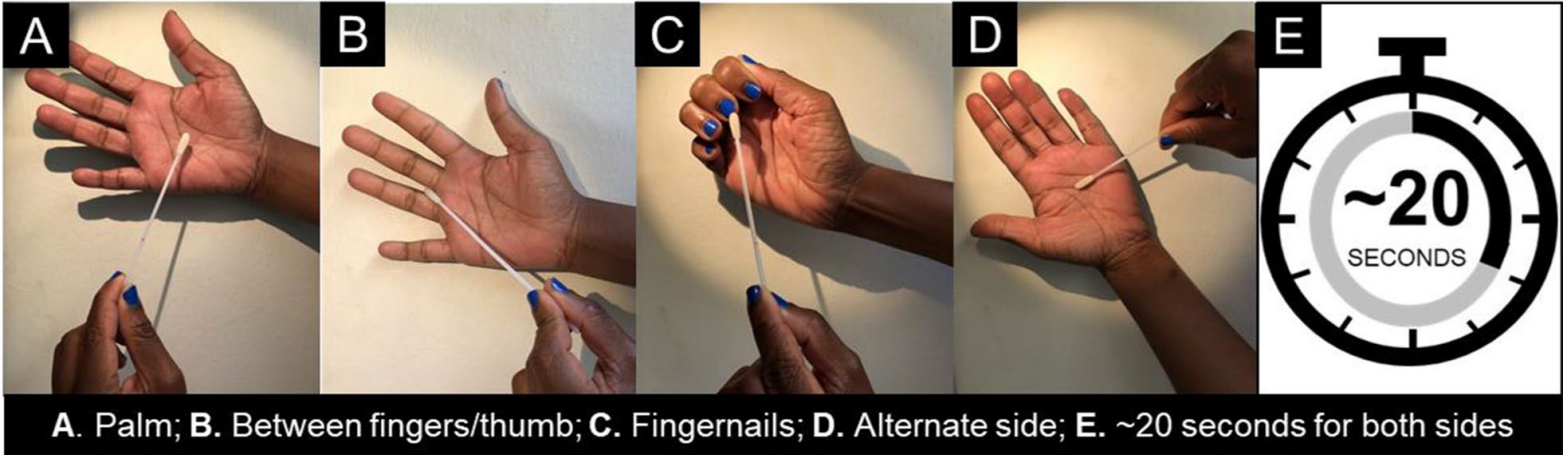
Hand self-swab technique used by caregivers and healthcare workers

**Fig. 3:**
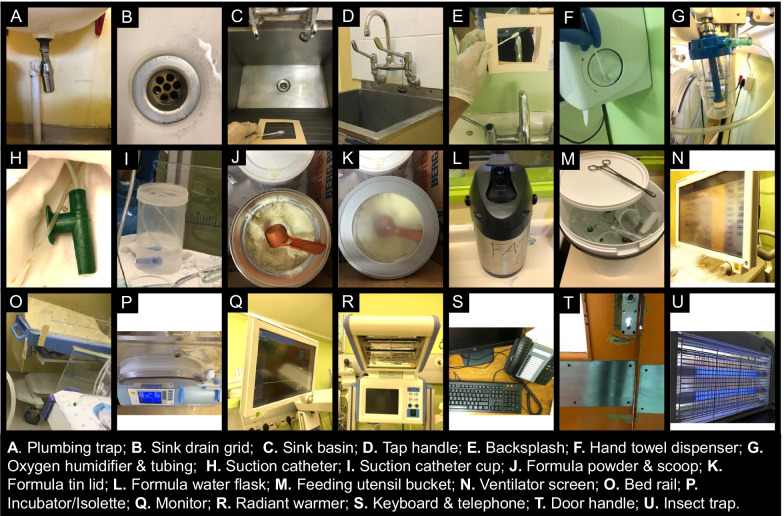
Sites sampled during environmental sampling in the neonatal unit

- **Sampling devices**
  We used sampling equipment that was relatively inexpensive and easy to source using local vendors in our setting (Fig. 1). This included: 15 cm sterile nylon flocked swabs with solid plastic handle and breakpoint (Puritan®; Cat. No. 25-3406-U, a 10 cm × 10 cm paper single-use paper sampling template for flat surface sampling (Environmental Express®; Cat. No. EE-C1010), 15 ml plastic conical centrifuge tubes (SPL Life Sciences; Cat. No. 51015) for water samples and for transport of flocked swab samples, a 100 ml sterile syringe and tubing (CareJoy, Cat. No. 784121) for water aspiration, and nitrile or latex examination gloves for the person collecting the sample.
- **Sample preparation & transport**
  Prior to sample collection, flocked swabs were pre-moistened with 1 ml of sterile normal saline at room temperature which had been inoculated into the conical tubes. After sample collection, water samples and flocked swab samples were placed in the conical tubes and transported in an ice pack and cooler box to the laboratory, kept at 4^o^ degrees Celsius until being processed within 24–48 h.
- **Sample collection technique**
  i. **Water**
    Free-flow water samples were collected directly from taps after allowing water to run for 5 s. Water samples taken from plumbing traps were obtained by inserting a 4 mm diameter sterile plastic hose (alternatively, a naso-gastric tube can also be used) into the sink grid and water aspirated into a 100 ml sterile syringe (smaller syringes can also be used).
  ii. **Flat surfaces**
    A 10 cm × 10 cm single-use paper sampling template was placed on a flat surface and a pre-moistened flocked swab was streaked in a zigzag motion in three different orientations, rotating the swab while streaking for a total of 20 s.
  iii. **Non-flat surfaces**
    A pre-moistened flocked swab was streaked in a zigzag motion in and around the non-flat surface, rotating the swab while streaking for a total of 20 s.
  iv. **Infant formula**
    A pre-moistened, sterile flocked swab was stirred into an opened powered infant formula tin for approximately five seconds and placed into a conical tube.
  v. **Trapped insects**
    Insects were trapped in the immediate patient care environment using an LED light insect trap already in use in the unit to control insects. Insect corpses were lifted into the conical tubes using flocked swabs.
  vi. **Hands**
    Voluntary, anonymous swabbing of hands of healthcare workers and caregivers was carried out during three of the four sampling events. Healthcare workers and caregivers were asked to self-swab their hands using a standard technique illustrated in Fig. 2 prior to washing their hands with soap, water, and then drying and sanitizing with an alcohol-based hand rub (ABHR). Hand swabs were taken in the same manner after handwashing. In this neonatal unit where, prior to the Corona Virus Disease 2019 (COVID-19) pandemic, ABHR was not always available, handwashing upon entry to the unit using soap and water is enforced per hospital infection control guidelines. However, per World Health Organisation Guidelines on Hand Hygiene in Health Care, all hand hygiene should be performed using ABHR; the soap-and-water method should be employed only when hands are visibly soiled or after using the toilet.[16].
- **Sites sampled**
  We sampled a combination of water, high-touch surfaces, infant formula, medical equipment, and trapped insects recovered from the immediate patient care area (Fig. 3). The same sites were sampled on all four occasions, however some sampling sites were added and removed as the sampling strategy was refined over time, and thus not all sites have four sampling results.
- **Sample processing and culturing**
  Swabs and saline-containing tubes were shaken on a vortex mixer to elute the sampled organisms and directly inoculated onto chromogenic-agar-media (CHROMagar™ ESBL, Paris, France), which is selective and differential for ESBL, using a 10 µL graduated inoculating loop, streaking the plates to isolate individual colonies. Bacterial colonies were quantified and phenotypically characterized using biochemical identification tests. No growth was defined as growth of < 10 colony-forming units (CFUs); light bacterial growth was defined as growth of 10 –100 CFUs, growth of > 100 – < 1000 CFUs was considered moderate growth, and > 1000 CFUs was considered heavy growth. For flat surfaces, bacterial growth was categorized using these CFU breakpoints per 100 cm^2^.
- **Data analysis**
  Data were analysed using basic descriptive statistics: crude numbers and frequencies using Microsoft Excel Software.

## Results

In total, 567 samples were collected, 248 (44%) of which grew ESBL, including species *Klebsiella* spp., *Acinetobacter* spp., *Pseudomonas* spp., *Enterobacter* spp., *Citrobacter* spp., and *Escherichia coli*.

**Fig. 4:**
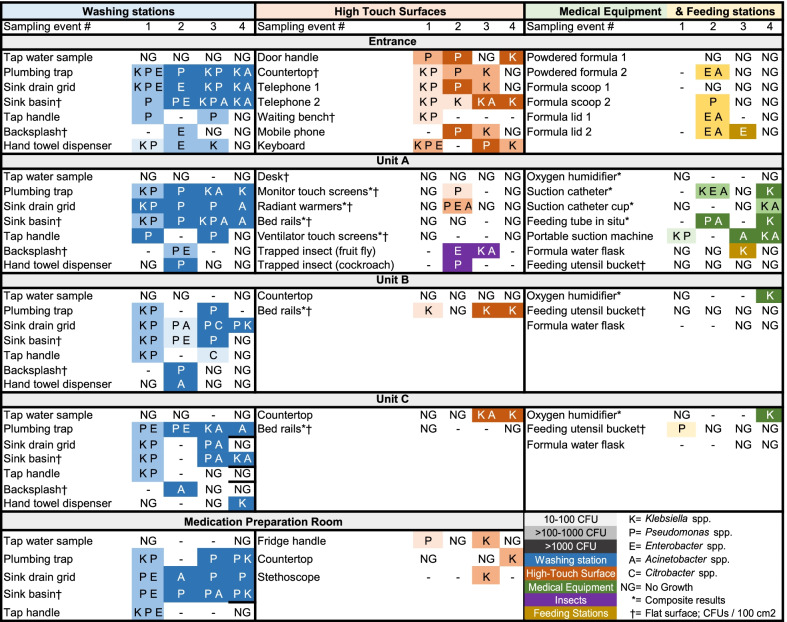
Contamination by extended-spectrum beta-lactamase producing organisms, by location, sample type, sampling event

- **Results from environmental samples**
  Dense and consistent ESBL contamination was detected inside plumbing traps, sink drain grids, and sink basins at all locations within the unit during all four sampling events (Fig. 4). Contamination was also consistently observed in certain high-touch surfaces (telephones, door handles, keyboards), while contamination was intermittently demonstrated on medical equipment (suction catheters, feeding tubes, and oxygen humidifiers) and feeding stations (including formula powder). Free-flow samples from tap water consistently demonstrated no growth.
- **Results from hand swabs**
  Hand contamination rates were higher among caregivers than among healthcare workers (Table 1). The most commonly identified organisms recovered from hands of healthcare workers were *Pseudomonas* spp., accounting for 42% of isolates recovered, followed by *Acinetobacter spp.* (30%), *Klebsiella* spp. (15%), *Enterobacter* spp. (12%), and *E.coli* (4%). Among caregivers, the most commonly identified organisms recovered from hand cultures were *Klebsiella* spp. (38%), *Acinetobacter spp.* (38%), *Enterobacter* spp. (11%), *Pseudomonas* spp. (9%), and *E. coli* (4%). Microbial contamination declined during all sampling events after washing, except among healthcare workers during the first sampling event, when contamination *increased* from 29 to 38% after washing, with most hands testing positive for *Pseudomonas* spp. Following these results, all participants were instructed to use ABHR following washing with soap and water.
  Of note, bacterial genera were recovered with roughly equal distribution over time, with the exception of *Acinetobacter* spp., which was not recovered at all during the first sampling event in January, but from 16 and 21 diverse sampling sites during the 2nd and 3rd sampling events. The third sampling event corresponded with an increase in *Acinetobacter* infections hospital wide, as per microbiological blood culture surveillance.
- **Feasibility and Efficiency Assessment**
  Most supplies were sourced using local vendors. To collect specimens, plate, and analyse 50 samples, we estimated a total expenditure of $269.40 United States Dollars and 13.5 cumulative work hours among all personnel. Culture results were available within 24–72 h of collection. Although these results were analysed by a clinical microbiologist, the processing and culturing techniques were deemed to be appropriate for the level of a microbiology lab technician.

**Table 1:** ESBL contamination of hands pre- and post-handwashing, by healthcare worker or caregiver, and sampling event

	Sampling event #1	Sampling event #2	Sampling event #3
*Healthcare worker hands*			
Pre-wash contaminated	29% (7/24)	40% (2/5)	45% (5/11)
Post-wash contaminated	38% (9/24)*	20% (1/5)	18% (2/11)
*Caregiver hands*			
Pre-wash contaminated	85% (11/13)	55% (11/20)	63% (5/8)
Post-wash contaminated	55% (6/11)^†^	53% (8/15) ^†^	38% (3/8)

^*^Contamination among healthcare workers increased during the first sampling event, with *Pseudomonas* spp. accounting for most post-wash cultures

^†^Some participants were lost to follow-up to obtain post-wash swabs

## Discussion

Using basic environmental sampling and laboratory techniques aided by chromogenic culture media, we identified ESBL reservoirs (sinks) and plausible transmission vehicles (medical equipment, infant formula, hands of caregivers/healthcare workers, insects) in this NICU environment. This strategy was a simple and cost-efficient method to assess ESBL bioburden and may be feasible for use in other settings to support ongoing infection control assessments and outbreak investigations.

The identification of sinks as stable reservoirs for ESBL in this setting was anticipated; the moist and warm environments created by plumbing traps provide ideal conditions for biofilm formation [17]. Furthermore, eradication of ESBL from plumbing traps using conventional remediation techniques is challenging and therefore allows for long-term survival of bacterial communities [18]. Additionally, the stable contamination of sink grids, basins, tap handles, and some backsplashes demonstrated in this study illustrates the retrograde model of bacterial dispersal from wastewater previously postulated by in situ studies [17]. The fact that some healthcare workers’ hands demonstrated *Pseudomonas* spp. post-handwashing which were not present prior to handwashing is concerning for acquisition from the sink environment (although could be explained by variability of swab technique). Following these results, the importance of using ABHR for all hand hygiene (including after washing with soap and water) was re-emphasized to all staff and caregivers.

The recognition of sinks as environmental reservoirs for ESBL has led some intensive care units (ICUs) to adopt “water free” care, moving sinks away from the immediate patient care environment, with corresponding declines in patient colonization rates for pathogens of interest [19]. In settings where these changes may not be feasible, interim measures targeting transmission vehicles are appropriate. In this study, we identified medical equipment, formula, insects, and hands of healthcare workers and caregivers as potential transmission vehicles that might move pathogenic organisms from a fixed location to the patient. Infection control teams should engage with doctors, nurses, and cleaning staff to ensure shared medical equipment is adequately disinfected in between uses, according to equipment package inserts or best practices, such as the U.S. Centers for Disease Control and Prevention’s “Environmental Cleaning in Healthcare Facilities in Resource-Limited Settings” [20]. Preparation of shared powdered infant formula sources should be carefully monitored and, if possible, carried out in accordance with World Health Organisation’s guidelines for safe preparation, storage and handling of powdered infant formula [21].

We found that insects within the patient care environment were often colonized with potentially pathogenic organisms. While insect infestations are not typically reported as contributing to either endemic or outbreaks of healthcare-associated infections, the importance of vector control in the patient care environment cannot be over-emphasized. For example, cockroaches have been implicated in outbreaks of neonatal *K. pneumoniae* infections [22], and ESBL colonization of fruit flies is well-documented [23]. In our study setting, LED-light insect traps have proved to be an effective measure, along with window screens, at reducing the presence of insects in the patient care environment.

There are several limitations to this study, both in its technical and conceptual approach. First, environmental sampling was conducted at a single centre, and findings may not be generalizable to other units, depending on local climate, patient census, and cleaning practices. These point prevalence surveys were conducted in a ward that was actively used for patient care and the timing of sample collection had to adjust to the demands of patient care. Our sampling events were not coordinated with cleaning times, so we are unable to determine the extent to which current cleaning practices were effective in reducing the burden of contamination. We attempted to overcome this limitation by performing multiple sampling events and categorizing single contaminations as “transient” and repeated contaminations as “stable”. Our use of “semi-sterile” techniques could have resulted in some false-positive results due to inadvertent contamination, but again we sought to counteract these limitations through the use of serial sampling. Although the chromogenic media chosen purports inhibition of all non-ESBLs, including those producing ampC beta lactamases, the possibility of false positive results have been noted by other studies [24]. Thus, an overestimation of ESBL bioburden remains until isolates are confirmed using standard antimicrobial sensitivity methods.

Sampling bias may have influenced our findings on the prevalence of hand carriage of potential pathogens by caregivers and healthcare workers. For example, those confident with their hand hygiene practices may have been more willing to participate, leading to an under-estimation of the true contamination rate.

This study took place in the midst of the COVID-19 pandemic, when heightened efforts to mitigate hospital transmission of Severe Acute Respiratory Syndrome Coronavirus-2 (SARS-CoV-2) were in place. In comparison to pre-pandemic practices, hand hygiene was enforced more strictly and soap and ABHR were more readily available. These important measures may have contributed to an overall decreased bacterial bioburden, particularly on the hands of healthcare workers and caregivers.

We are unable to confirm that the identified environmental contamination contributed to concurrent clinical infections. In future studies, whole genome sequencing can help to establish true transmission pathways.

Despite these limitations, this simple environmental sampling technique might be a feasible way in which neonatal and other high-risk units facing hyper-endemic rates of ESBL infections can better understand the nature of contamination and transmission dynamics in their unit. Further, it might help catalyse the implementation of infection control measures targeting defined reservoirs and suspected transmission pathways. The temporal link between increased recovery of *Acinetobacter* spp. from the environment and increased incidence of *Acinetobacter* infections hospital-wide is an anecdote of the importance of timely and reliable environmental sampling as an important outbreak response tool. This sampling method may also be pivotal in trialling and measuring the impact of novel remediation and prevention strategies. For example, neonatal units who have identified sinks as stable ESBL reservoirs should consider removing sinks and implementing “water-free” care for ICU patients [19], placing sink covers [25], or implementing anti-microbial alloys, such as copper, which has been shown to reduce microbial burden in NICUs [26]. This environmental sampling technique can be used to test microbial burden before and after implementation of these infection control interventions and track bioburden overtime.

## Conclusion

In conclusion, basic environmental sampling and laboratory techniques aided by chromogenic culture media are sufficient to identify ESBL reservoirs and transmission vehicles and may be used in similar settings to support ongoing infection control assessments and outbreak investigations. More research is needed to understand the role of the environment in influencing risk of ESBL colonization and disease in neonates.

## Data Availability

All data generated or analysed during this study are included in this published article.

## Abbreviations

ABHR: Alcohol-Based Hand Rub
COVID-19: Corona Virus Disease 2019
CFU: Colony-forming units
ESBL: Extended-Spectrum Beta-Lactamase Producing Organism
ICU: Intensive Care Unit
LED: Light Emitting Diode
NICU: Neonatal Intensive Care Unit
SARS-CoV-2: Severe Acute Respiratory Syndrome Coronavirus-2

## Footnotes

1 For clinical reports from Botswana, extended-spectrum beta-lactamase production is inferred from extended-spectrum cephalosporin resistance detected on phenotypic antibiotic susceptibility testing.
